# Evidence of multisensory plasticity: Asymmetrical medial geniculate body in people with one eye

**DOI:** 10.1016/j.nicl.2015.09.016

**Published:** 2015-10-09

**Authors:** Stefania S. Moro, Krista R. Kelly, Larissa McKetton, Brenda L. Gallie, Jennifer K.E. Steeves

**Affiliations:** a Department of Psychology, York University, Toronto, Canada; b Centre for Vision Research, York University, Toronto, Canada; c Department of Ophthalmology and Visual Sciences, The Hospital for Sick Children, Toronto, Canada; d Department of Biology, York University, Toronto, Canada; e Retina Foundation of the Southwest, Dallas, TX, USA

**Keywords:** Monocular enucleation, Medial geniculate body, Audiovisual processing, Subcortical reorganization, Plasticity, Multisensory

## Abstract

The medial geniculate body (MGB) plays a central role in auditory processing with both efferent and afferent tracts to primary auditory cortex. People who have lost one eye early in life have enhanced sound localization, lack visual over auditory dominance and integrate auditory and visual information optimally, similar to controls, despite taking longer to localize unimodal visual stimuli. Compared to controls, people with one eye have decreased lateral geniculate nuclei (LGN) volume as expected given the 50% deafferentation of the visual system. However, LGN volume is larger than predicted contralateral to the remaining eye, indicating altered structural development likely through recruitment of deafferented LGN cells.

*Purpose*: the current study investigated whether structural MGB changes are also present in this group given the changes they exhibit in auditory processing.

*Methods*: MGB volumes were measured in adults who had undergone early unilateral eye enucleation and were compared to binocularly intact controls.

*Results*: unlike controls, people with one eye had a significant asymmetry with a larger left compared to right MGB, independent of eye of enucleation. MGB volume correlated positively with LGN volume in people with one eye.

*Conclusions*: volume asymmetry in the MGB in people with one eye may represent increased interactions between the left MGB and primary auditory cortex. This interaction could contribute to increased auditory and other left hemisphere-dominant processing, including language, as compensation for the loss of one half of visual inputs early in life. The positive correlation between MGB and LGN volume is not due to space constraints but rather indicates increased plasticity in both auditory and visual sensory systems following early eye enucleation.

## Highlights

• We measured MGB volume in people with one eye with high resolution MRI.
• People with one eye had significantly larger left compared to right MGB volume.
• May indicate increased interactions between left MGB and primary auditory cortex.
• Positive correlation of left MGB with LGN volume indicates cross-sensory plasticity.
• Increased left hemisphere processing may compensate for the loss of one eye.

## 1 Introduction

Every day, as we interact with the world, we take in important information from the environment through our sensory systems. Sensory information, with the exception of olfaction, is first processed subcortically by the thalamus, which then projects information to the cortex for further processing (Bartlett, 2013). There is evidence that when one sense is compromised, as in the case of complete blindness, brain regions associated with the lost sense (i.e., visual cortex) can be reorganized for use by the other senses (e.g., Röder et al., 2002). We have recently shown that structural reorganization at the subcortical level in the lateral geniculate nucleus (LGN) of the thalamus occurs in cases of sensory deprivation (Kelly et al., 2014).

The medial geniculate body (MGB) of the thalamus plays a central role in auditory processing (Devlin, 2006; Bartlett, 2013). It relays auditory information for higher order processing in primary auditory cortex, however, substantial reciprocal connections from the primary auditory cortex back to the MGB also exist (Bartlett, 2013; Lee et al., 2004; Lee, 2013). This combination of ascending/descending sensory connections allows for the complex perception of sounds (Bartlett, 2013). In humans, functional magnetic resonance imaging (fMRI) has shown activation in the MGB during sound localization and sound recognition (Maeder et al., 2001), as well as speech and emotional voice discrimination (von Kriegstein et al., 2008; Ethofer et al., 2012). Cortical feedback can alter MGB responses and provide dynamic gain enhancement or suppression through direct excitatory (corticothalamic feedback) or indirect inhibitory (corticothalamic influence on the thalamic reticular nucleus (TRN)) signals (Zhang et al., 1997; Bartlett, 2013). This gain control may contribute to enhancing auditory attention and context memory, important for predicting words in a spoken sentence (Bartlett, 2013).

Extensive investigation of the mammalian MGB, typically in the cat, has revealed three major subdivisions: ventral (vMGB), medial (mMGB) and dorsal (dMGB) (Devlin, 2006). The ventral division is sensitive to pure tone stimulation of the contralateral ear, whereas both the medial and dorsal divisions are more sensitive to complex and multisensory stimuli (see De Ribaupierre, 1997; Rouiller, 1997 for review). In addition, non-auditory stimuli also activate the MGB. The rat MGB responds to light flashes or rewards indicating that it plays a role in integrating multisensory stimuli to provide an enhanced contextual response (Komura et al. 2001, Komura et al. 2005; Bartlett, 2013). Congenitally deaf mice show reorganization at the level of the thalamus through the activation of the MGB (Hunt et al., 2005). Together, these findings indicate that it is possible for primary sensory afferents, such as retinal projections, to rewire and claim unused subcortical structures, resulting in anatomical re-modelling (Hunt et al., 2005; Karlen, 2006). Reorganization of the MGB following sensory loss, however, has not been studied extensively in humans.

Monocular enucleation, the surgical removal of one eye early in life, is a unique form of visual deprivation that provides a useful model for studying and quantifying the underlying neural consequences of the loss of binocularity during development. Monocular enucleation differs from other more common forms of monocular deprivation, such as strabismus and amblyopia, by providing a clean model of total monocular deprivation. This is unlike strabismus and amblyopia which result in unreliable, unbalanced competing visual signals from the deprived eye. Monocular enucleation results in only one stream of normal visual input from the remaining eye to the visual system. Monocular enucleation also provides an excellent model for investigating the interaction between vision and other senses, since vision has not been completely eliminated (see Kelly et al., 2013; Steeves et al., 2008 for reviews).

It has been well documented that the visual system changes in response to the loss of one eye. People who have had one eye removed early in life when the visual system is not yet mature demonstrate altered processing in their remaining senses. For example, they show intact (Cattaneo, 2014) or enhanced visual spatial form ability (Nicholas, Heywood, and Cowey 1996, Reed et al., 1997, Steeves et al., 2004), but reduced visual motion processing (see Steeves et al., 2008; Kelly et al., 2013 for reviews). Some evidence of cross-modal adaptation in response to the compromised enucleated visual system has also been demonstrated. People who lost an eye early in life show enhanced auditory localization in the horizontal azimuth compared to binocular controls (Hoover et al., 2012). Further, they do not show the typical pattern of visual over auditory dominance (i.e., Colavita visual dominance effect; Colavita, 1974) that binocular controls exhibit, but rather show equivalent auditory and visual processing (Moro and Steeves, 2012). Lastly, although people with one eye do not integrate audiovisual stimuli any differently from controls during audiovisual spatial localization, they are slower to respond when localizing visual stimuli (Moro et al., 2014).

There have been few studies investigating the morphology of sensory systems of people who have lost an eye early in life (Kelly et al., 2014; Kelly et al., 2015). Subcortically, people with one eye have an overall decrease in LGN (the visual relay station of the thalamus) volume compared to binocular viewing controls (Kelly et al., 2014). This is not surprising given the 50% deafferentation of signal to the visual system with eye enucleation. What is surprising, however, is that the LGN contralateral to the remaining eye is less reduced in volume likely from recruitment of some of the deafferented LGN cells (Kelly et al., 2014). This finding provides evidence that the visual system, even at the level of the subcortex, is vulnerable to reorganization after losing one eye early in life during the period of normal maturation (Kelly et al., 2014).

Functional neuroimaging can be used to successfully localize the MGB in humans, however, this methodology is limited by sensory stimuli and in its sensitivity (Devlin, 2006). Devlin et al. (2006) have been able to structurally image the MGB to resolve the functional localization concerns. Their method is now considered the gold standard for anatomical localization of the MGB and other thalamic structures. Given the existing auditory and audiovisual behavioural differences and the morphological changes in the LGN in people with one eye, we investigated whether structural changes in the MGB also exist in this group.

## 2 Methods

### 2.1 Participants

#### 2.1.1 People with one eye (monocular enucleation, ME)

Ten adult participants who had undergone monocular enucleation (ME) at The Hospital for Sick Children participated in this study (mean age = 26 years, SD = 10; 5 female). All ME participants had been unilaterally eye enucleated (5 right eye removed) due to retinoblastoma, a rare childhood cancer of the retina. Age at enucleation ranged from 4 to 60 months (mean age at enucleation = 21 months, SD = 16).

#### 2.1.2 Binocular viewing control participants (BV)

Fifteen binocularly intact controls with a mean age of 30 years (SD = 11; 6 female; 11 right eye dominant) were tested and reported no history of abnormal visual experience.

All participants (ME, BV) reported normal hearing, normal or corrected-to-normal acuity as assessed by an EDTRS eye chart (Precision Vision™, La Salle, IL) and wore optical correction if needed. All participants gave informed consent prior to inclusion in the study, which adhered to the tenets of the Declaration of Helsinki and was approved by the York University Office of Research Ethics.

### 2.2 Data acquisition, processing and measurements

All scans were acquired on a Siemens MAGNETOM Trio 3 T MRI scanner with a 32-channel head coil in the Sherman Health Sciences Research Centre at York University. Proton density (PD) weighted images were processed using tools from the freely available FMRIB's Software Library (FSL; version 4.1.8) (http://www.fmrib.ox.ac.uk/fsl.) All data acquisition and processing were conducted according to methods used in Kelly et al. (2014) for assessing LGN volume. Thalamic nuclei have been successfully identified using PD weighted images previously (Devlin et al., 2006; Kelly et al., 2014). See Kelly et al. (2014) for more detailed data acquisition and processing procedures.

High-resolution T_1_ weighted images were acquired with the following parameters: rapid gradient echo, 1 mm^3^ isotropic voxels, TR = 1900 ms, TE = 2.52 ms, 256x256 matrix, and flip angle = 9°. Either 30 or 40 PD weighted images per participant were acquired coronally with the following parameters: turbo spin echo, 800 x 800 µm in-plane resolution, slice thickness = 2 or 1 mm, TR = 3000 ms, TE = 22 or 26 ms, 256x256 matrix, and flip angle = 120°. Total scan time per participant was approximately 1.5 h. The smaller number (30) of PD weighted images for some participants was due to time constraints.

Using FSL toolbox applications, all PD weighted images for each participant were interpolated to twice the resolution and half the voxel size using FLIRT (Jenkinson and Smith, 2001; Jenkinson et al., 2002) to increase the signal-to-noise ratio. These images were then concatenated using fslmerge and motion-corrected using MCFLIRT (Jenkinson et al., 2002). From the series of interpolated PD weighted images, a mean high resolution PD image was created per participant using fslmaths.

**Fig. 1:**
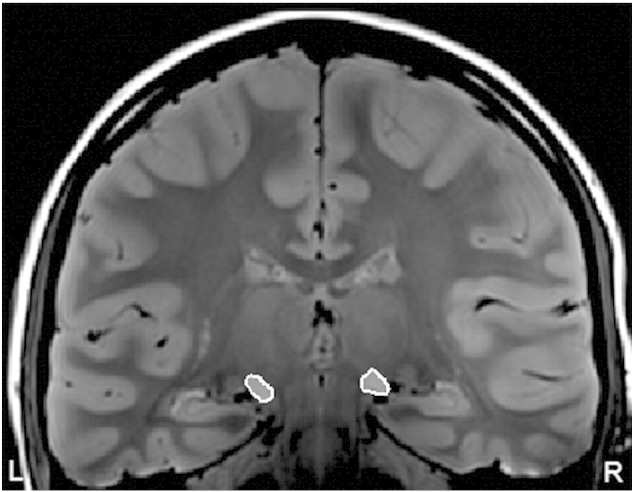
A: An averaged, interpolated PD weighted image of a typical control participant indicating the final median ROI of left and right MGB mask outlined in white.

Following image acquisition and processing, three independent raters manually traced the left and right MGB region of interest (ROI) masks three times each for each participant using the mean PD weighted image. For each rater, ROIs were merged and a median mask was created using fslmerge and fslmaths, respectively. Median masks from each of the three raters were merged together and a final median mask across raters was created. MGB volumes (left and right) were calculated for each participant from this final median mask using fslstats. All intra-rater inter-class correlations (ICC) were above 0.85 and all inter-rater ICCs were above 0.85. ICCs above 0.70 indicate that measurements were consistent both within and between raters (Cohen, 2001). These methods are the gold standard and most appropriate for evaluating thalamic structures in clinical settings (e.g., Bridge et al., 2009; Devlin, 2006; Schmitz et al., 2003). An averaged, interpolated PD weighted image with an outline of the final median ROI left and right MGB mask is shown in Fig. 1.

## 3 Results

### 3.1 Ipsilateral vs contralateral MGB volume

**Fig. 2:**
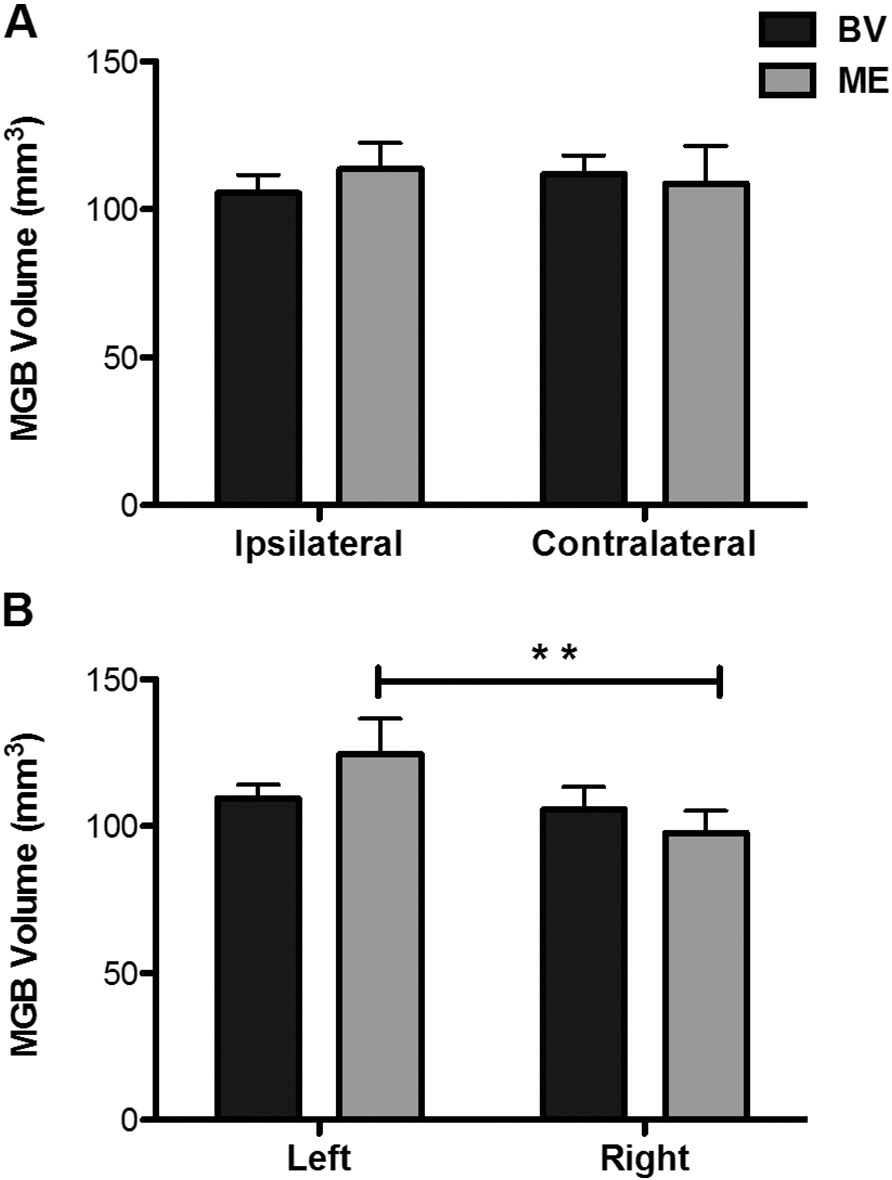
A. MGB volume (mm^3^) in the ipsilateral and contralateral hemisphere to the dominant or remaining eye of BV (black) and ME (grey) groups, respectively. B. MGB volume in the left and the right hemisphere of the BV and ME groups.

A 2 × 2 repeated measures analysis of variance (ANOVA) comparing group (ME vs BV) and MGB side (ipsilateral vs contralateral to remaining or dominant eye) revealed no significant interaction, *F*(1,23) = 0.825, *p* = 0.373 and ŋ_p_^2^ = 0.035. There was no main effect of participant group, *F*(1,23) = 0.059, *p* = 0.810 and ŋ_p_^2^ = 0.003, or MGB side, *F*(1,23) = 0.013, *p* = 0.909 and ŋ_p_^2^ = 0.001. People with one eye did not differ in MGB volume compared to controls. Fig. 2a plots MGB volume ipsilateral and contralateral to the dominant or remaining eye of the BV and ME groups respectively.

### 3.2 Left vs right MGB volume

A 2 × 2 repeated measures ANOVA comparing participant group (ME vs BV) and MGB side (left vs right) revealed a trend toward an interaction, *F*(1,23) = 4.159, *p* = 0.053 and ŋ_p_^2^ = 0.153. There was no main effect of participant group, *F*(1,23) = 0.059, *p* = 0.810 and ŋ_p_^2^ = 0.003, but there was a significant main effect of MGB side, *F*(1,23) = 10.879, *p* = 0.003 and ŋ_p_^2^ = 0.321. Bonferroni corrected post-hoc comparisons revealed that people with one eye had a significantly larger left compared to right MGB volume (*p* = 0.002), regardless of which eye was removed. This asymmetry was not present in the BV group (*p* = 0.330). Fig. 2b plots the left and the right MGB volume of the BV and ME groups.

### 3.3 Comparing LGN and MGB volumes

**Fig. 3:**
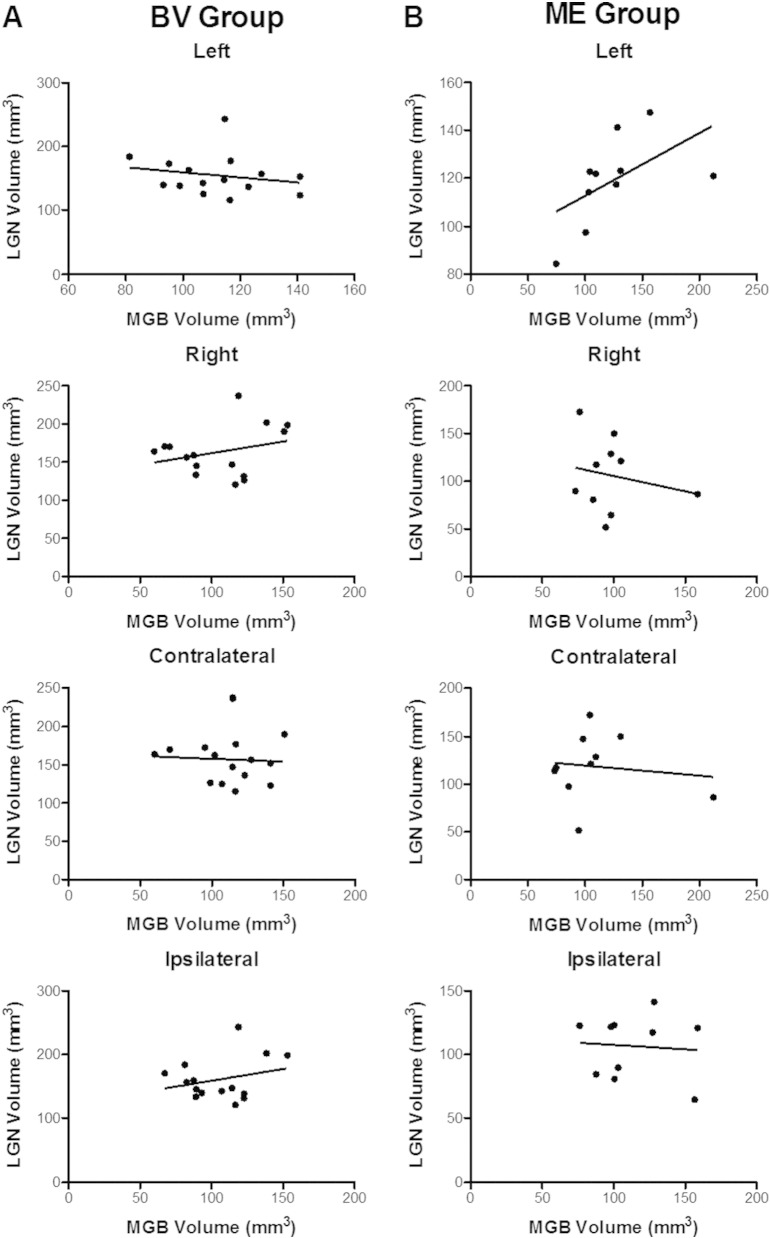
MGB volume (mm^3^) correlated with LGN volume (mm^3^) (taken from Kelly et al., 2014) for control participants (Column A) and for the ME group (Column B).

We investigated the relationship between the existing LGN volumes (Kelly et al., 2014) and our current MGB volumes. For our control participants non-parametric Spearman correlations comparing left MGB to left LGN, *r*(15) = −0.25, *p* = 0.36 and right MGB to right LGN, *r*(15) = 0.13, *p* = 0.64 were not significant. For our ME group, there is a strong positive Spearman's correlation comparing left MGB to left LGN, *r*(10) = 0.73, *p* = 0.02, while there is no significant correlation when comparing the right MGB to right LGN volumes, *r*(10) = −0.03, *p* = 0.95. Furthermore, there were no significant correlations when comparing the contralateral (to the dominant or remaining eye of participants) LGN to the contralateral MGB in both controls: *r*(15) = −0.11, *p* = 0.71 and ME group: *r*(10) = 0.28, *p* = 0.43, or when comparing ipsilateral LGN to ipsilateral MGB in both controls: *r*(15) = −0.02, *p* = 0.96 and ME group: *r*(10) = −0.16, *p* = 0.66. Fig. 3A plots the LGN and MGB correlations for the control participants. B: plots the LGN and MGB correlations for the ME group.

## 4 Discussion

The current study used structural MRI to anatomically localize and measure the MGB volume in people with one eye and binocularly intact controls. Overall, people with one eye displayed an asymmetry in MGB volume with a larger left than right MGB, regardless of which eye was enucleated. Binocularly intact controls did not display an asymmetry.

Although traditionally viewed as an audiocentric structure, rat studies have shown that subnuclei of the MGB also receive some visual input (Linke et al., 2000; Komura et al., 2001, 2005). In humans, participants presented with low intensity visual stimuli paired with an auditory tone show increased behavioural sensitivity and functional activation in the MGB, indicating crossmodal influences on the MGB (Noesselt et al., 2010). These crossmodal influences on the MGB might be driven through corticothalamic feedback mechanisms, which in turn may influence multisensory interactions (Noesselt et al., 2010). In addition, people with one eye have superior sound localization abilities (Hoover et al., 2012) supporting the documented coding of sound localization in the MGB (Samson et al., 2000). Given that our patient population is missing half of the retinal inputs to the visual system, the MGB asymmetry observed in people with one eye may reflect neural reorganization at the subcortical level during postnatal maturation. This is consistent with the cortical changes that have been found in this patient group described below.

Recently, Kelly et al. (2015) found that people with one eye exhibited significantly larger cortical surface area in auditory supramarginal and superior temporal regions, specifically in the left hemisphere compared to binocularly intact controls. These regions are implicated in short-term memory of auditory information (Paulesu et al., 1993) and audiovisual multisensory integration (Beauchamp et al., 2004), respectively, indicating reorganization in cortical areas outside of primary visual regions following early monocular enucleation (Kelly et al., 2015). The cortical surface area, LGN and the present findings complement auditory and audiovisual behavioural data in this monocular enucleation group showing equal processing of paired auditory and visual signals (Moro and Steeves, 2012), better sound localization (Hoover et al., 2012) and optimal audiovisual integration (Moro et al., 2014) compared to controls. It is possible that the increase in cortical surface area in the left hemisphere auditory and multisensory regions with monocular enucleation is reflected in strengthened corticothalamic feedback to the left MGB and has lead to the presently observed MGB volume asymmetry.

The relationship between the previously published LGN and the present MGB volumes in both controls and people with one eye were correlated. If the larger MGB volume were limited by space constraints then the MGB asymmetry should be related to a decrease in size of the LGN and one would predict a negative correlation. We found no relationship in any comparison except for a strong *positive* correlation between the left MGB and left LGN in people with one eye. This signifies that a larger left MGB is related to a larger left LGN in people with one eye and rules out the space constraints prediction. A larger left MGB cannot be solely explained as a harvesting of unused subcortical real estate since a larger left MGB is associated with a larger left LGN. Instead, a strong positive correlation indicates overall increased plasticity across sensory systems that is not restricted by subcortical space confines but rather is related to corticothalamic feedback from left hemisphere cortical areas that have larger surface area (Kelly et al., 2015) thereby demonstrating multi-level reorganization of sensory systems.

Multisensory events, such as audiovisual speech processing, have been reported to involve thalamic modulation (Baier et al., 2006; Cappe et al., 2009; Musacchia et al., 2007) and the MGB responds preferentially to more complex speech-like structure (Jiang et al., 2013). People with dyslexia show a reversed hemispheric asymmetry to our current findings (i.e., smaller left versus right MGB) (Galaburda, 1994). The left hemisphere of cortex plays a central role in language processing due to its analysis of fast temporal auditory transitions (Galaburda, 1994). Phonological abnormalities in people with dyslexia may be reflected in adverse left hemisphere cortical reorganization contributing to the reverse MGB asymmetry found in dyslexia (Galaburda, 1994). People with one eye have mild face recognition deficits compared to binocular and monocular viewing controls (Kelly et al., 2012). It is possible that as a consequence of their mild face-processing deficit, the brain of people with one eye has reorganized to emphasize speech and language for identifying individuals, which could lead to specific structural changes to language areas in the left hemisphere of the brain. Future studies investigating behavioural measures of speech and language processing in this group would be an ideal extension of these anatomical data.

## 5 Summary

The MGB volume asymmetry in people with one eye may represent increased interactions between the left MGB and primary auditory cortex. This interaction could contribute to increased auditory and other aspects of left hemisphere-dominant processing, including language. We observed overall multisensory plasticity with a relationship showing increasing plasticity in the visual LGN and the auditory MGB, reflecting subcortical reorganization as compensation for the loss of one half of visual inputs to the brain early in life.

## References

[bb0037] Baier B., Kleinschmidt A., Muller N.G. (2006). Cross-modal processing in early visual and auditory cortices depends on expected statistical relationship of multisensory information. J. Neurosci..

[bb001] Bartlett E.L. (2013). The organization and physiology of the auditory thalamus and its processing acoustic features important for speech perception. Brain Lang..

[bb0036] Beauchamp M.S., Argall B.D., Bodurka J., Duyn J.H., Martin A. (2004). Unraveling multisensory integration: patchy organization within human STS multisensory cortex. Nat. Neurosci..

[bb0030] Bridge H., Cowey A., Ragge N., Watkins K. (2009). Imaging studies in congenital anophthalmia reveal preservation of brain architecture in “visual” cortex. Brain.

[bb0038] Cappe C., Morel A., Barone P., Rouiller E.M. (2009). The thalamocortical projection systems in primate: an anatomical support for multisensory and sensorimotor interplay. Cereb. Cortex.

[bb0019] Cattaneo Z., Bona S., Monegato M., Pece A., Vecchi T., Herbert A.M., Merabet L.B. (2014). Visual symmetry perception in early onset monocular blindness. Vis. Cogn..

[bb0029] Cohen B. (2001). Explaining Psychological Statistics.

[bb0022] Colavita F.B. (1974). Human sensory dominance. Percept. Psychophys..

[bb0011] De Ribaupierre F., Ehret G., Romand R. (1997). Acoustical information processing in the auditory thalamus and cerebral cortex. The Central Auditory System.

[bb004] Devlin J.T., Sillery E.L., Hall D.A., Hobden P., Behrens T.E.J., Nunes R.G. (2006). Reliable identification of the auditory thalamus using multi-modal structural analyses. NeuroImage.

[bb009] Ethofer T., Bretscher J., Gschwind M., Kreifelts B., Wildgruber D., Vuilleumier P. (2012). Emotional voice areas: anatomic location, functional properties, and structural connections revealed by combined fMRI/DTI. Cereb. Cortex.

[bb0040] Galaburda A.M., Menard M.T., Rosen G.D. (1994). Evidence for aberrant auditory anatomy in developmental dyslexia. Proc. Natl Acad. Sci..

[bb0021] Hoover A.E.N., Harris L.R., Steeves J.K.E. (2012). Sensory compensation in sound localization in people with one eye. Exp. Brain Res..

[bb0015] Hunt D.L., King B., Kahn D.M., Yamoah E.N., Shull G.E., Krubitzer L. (2005). Aberrant retinal projections in congenitally deaf mice: how are phenotypic characteristics specified in development and evolution?. Anat. Rec..

[bb0028] Jenkinson M., Bannister P.R., Brady M., Smith S.M. (2002). Improved optimisation for the robust and accurate linear registration and motion correction of brain images. Neuroimage.

[bb0027] Jenkinson M., Smith S.M. (2001). A global optimisation method for robust affine registration of brain images. Med. Image Anal..

[bb0045] Jiang F., Stecker G.C., Fine I. (2013). Functional localization of the auditory thalamus in individual human subjects. NeuroImage.

[bb0016] Karlen S.J., Kahn D.M., Krubitzer L. (2006). Early blindness results in abnormal corticocortical and thalamocortical connections. Neuroscience.

[bb0025] Kelly K.R., DeSimone K.D., Gallie B.L., Steeves J.K.E. (2015). Increased cortical surface area and gyrification following long-term survival from early monocular enucleation. Neuroimage Clin..

[bb0041] Kelly K.R., Gallie B.L., Steeves J.K.E. (2012). Impaired face processing in early monocular deprivation from enucleation. Optom. Vis. Sci..

[bb003] Kelly K.R., McKetton L., Schneider K.A., Gallie B.L., Steeves J.K.E. (2014). Altered anterior visual system development following early monocular enucleation. Neuroimage Clin..

[bb0017] Kelly K.R., Moro S.S., Steeves J.K.E., Steeves J.K.E., Harris L.R. (2012). Living with one eye: plasticity in visual and auditory systems. Plasticity in Sensory Systems.

[bb0013] Komura Y., Tamura R., Uwano T., Nishijo H., Kaga K., Ono T. (2001). Retrospective and prospective coding for predicted reward in the sensory thalamus. Nature.

[bb0014] Komura Y., Tamura R., Uwano T., Nishijo H., Ono T. (2005). Auditory thalamus integrates visual inputs into behavioral gains. Nat. Neurosci..

[bb006] Lee C.C. (2013). Thalamic and cortical pathways supporting auditory processing. Brain Lang..

[bb005] Lee C.C., Schreiner C.E., Imaizumi K., Winer J.A. (2004). Tonotopic and heterotopic projection systems in physiologically defined auditory cortex. Neuroscience.

[bb0032] Linke R., Braune G., Schwegler H. (2000). Differential projection of the posterior paralaminar thalamic nuclei to the amygdaloid complex in the rat. Exp. Brain Res..

[bb007] Maeder P.P., Meuli R.A., Adriani M., Bellmann A., Fornari E., Thiran J.P. (2001). Distinct pathways involved in sound recognition and localization: a human fMRI study. Neuroimage.

[bb0023] Moro S.S., Steeves J.K.E. (2012). No Colavita effect: equal auditory and visual processing in people with one eye. Exp. Brain Res..

[bb0024] Moro S.S., Steeves J.K.E., Harris L.R. (2014). Optimal audiovisual processing in people with one eye. Multisens. Res..

[bb0039] Musacchia G., Sams M., Skoe E., Kraus N. (2007). Musicians have enhanced subcortical auditory and audiovisual processing of speech and music. Proceedings of the National Academy of Sciences.

[bb0020] Nicholas J.J., Heywood C.A., Cowey A. (1996). Contrast sensitivity in one-eyed subjects. Vision Research.

[bb0033] Noesselt T., Tyll S., Boehler C.N., Budinger E., Heinze H.-J., Driver J. (2010). Sound-induced enhancement of low-intensity vision: multisensory influences on human sensory specific cortices and thalamic bodies relate to perceptual enhancement of visual detection sensitivity. J. Neurosci..

[bb0035] Paulesu E., Frith C.D., Frackowiak R.S.J. (1993). The neural correlates of the verbal component of working memory. Nature.

[bb002] Röder B., Stock O., Bien S., Neville H., Rösler F. (2002). Speech processing activates visual cortex in congenitally blind humans. Eur. J. Neurosci..

[bb9995] Reed M.J., Steeves J.K.E., Steinbach M.J. (1997). A comparison of contrast letter thresholds in unilateral eye enucleated subjects and binocularly viewing control subjects.. Vis Res..

[bb0012] Rouiller E.M. (1997). Functional Organization of the Auditory Pathways.

[bb0034] Samson F.K., Barone P., Irons W.A., Clarey J.C., Poirier P., Imig T.J. (2000). Directionality derived from differential sensitivity to monaural and binaural cues in the cat's medial geniculate body. J. Neurophys..

[bb0052a] Schmitz B., Schaefer T., Krick C.M., Reith W., Backens M., Käsmann-Kellner B. (2003). Configuration of the optic chiasm in humans with albinism as revealed by magnetic resonance imaging. Invest. Ophthalmol. Vis. Sci..

[bb9910] Steeves J.K.E., Wilkinson F., González E.G., Wilson H.R., Steinbach M.J. (2004). Global shape discrimination at reduced contrast in enucleated observers. Vis. Res.

[bb0018] Steeves J.K.E., González E.G., Steinbach M.J. (2008). Vision with one eye: a review of visual function following monocular enucleation. Spat. Vis..

[bb0053] Zhang Y., Suga, N., Yan J. (1997). Corticofugal modulation of frequency processing in bat auditory system. Nature.

[bb008] von Kriegstein K., Patterson R.D., Griffiths T.D. (2008). Task-dependent modulation of medial geniculate body is behaviorally relevant for speech recognition. Curr. Biol..

